# The Value of Multiparametric Magnetic Resonance Imaging in the Preoperative Differential Diagnosis of Parotid Gland Tumors

**DOI:** 10.3390/cancers15041325

**Published:** 2023-02-19

**Authors:** Sebastian Stoia, Manuela Lenghel, Cristian Dinu, Tiberiu Tamaș, Simion Bran, Mihaela Băciuț, Emil Boțan, Daniel Leucuța, Gabriel Armencea, Florin Onișor, Grigore Băciuț

**Affiliations:** 1 Department of Maxillofacial Surgery and Implantology, Faculty of Dentistry, “Iuliu Hațieganu” University of Medicine and Pharmacy, 400012 Cluj-Napoca, Romania; 2 Department of Radiology, Faculty of Medicine, “Iuliu Hațieganu” University of Medicine and Pharmacy, 400012 Cluj-Napoca, Romania; 3 Department of Pathology, Emergency County Hospital, 600114 Cluj-Napoca, Romania; 4 Department of Medical Informatics and Biostatistics, “Iuliu Haţieganu” University of Medicine and Phamacy, 400012 Cluj-Napoca, Romania

**Keywords:** multiparametric magnetic resonance imaging, parotid gland, tumors, diffusion-weighted imaging, dynamic contrast enhanced

## Abstract

**Simple Summary:**

An accurate preoperative diagnosis of parotid gland tumors is of paramount importance in selecting the surgical strategy. The aim of the study was to determine the value of multiparametric MRI in the preoperative diagnosis of parotid tumors. We confirmed that T2WI and ADC showed statistically significant differences in the differential diagnosis of benign from malignant tumors. Multiparametric MRI showed a sensitivity, specificity, and accuracy of 81.8%, 88.6%, and 92.3%, respectively. All of the studied parameters were statistically significant in the differentiation of pleomorphic adenomas from Warthin tumors. With reference to the scope of this study, the conjunction of multiparametric and conventional MRI demonstrated a sensitivity, specificity, and accuracy of 94.1%, 100%, and 97.8%, respectively. Conventional MRl combined with diffusion-weighted imaging (DWI) and dynamic contrast–enhanced (DCE) multiparametric MRI improved the preoperative differential diagnosis of parotid gland tumors.

**Abstract:**

Background: The aim of the present study was to determine the value of multiparametric MRI in the preoperative differential diagnosis of parotid tumors, which is essential for therapeutic strategy selection. Methods: A three-year prospective study was conducted with 65 patients. Each patient was investigated preoperatively with multiparametric MRI and surgical excision of the tumor was performed. The preoperative imaging diagnosis was compared with the histopathological report. Several MRI parameters were analyzed, including T1 and T2 weighted image (WI), apparent diffusion coefficient (ADC), time to peak (TTP), and the time intensity curve (TIC). Results: In the differential diagnosis of benign from malignant tumors, T2WI and ADC showed statistically significant differences. Multiparametric MRI had a sensitivity, specificity, and accuracy of 81.8%, 88.6% and 92.3%, respectively. All of the studied parameters (T1, T2, TIC, TTP, ADC) were significantly different in the comparison between pleomorphic adenomas and Warthin tumors. With reference to the scope of this study, the conjunction of multiparametric and conventional MRI demonstrated a sensitivity, specificity, and accuracy of 94.1%, 100%, and 97.8%, respectively. Conclusions: Morphological analysis using conventional MRI combined with diffusion-weighted imaging (DW) and dynamic contrast–enhanced (DCE) multiparametric MRI improved the preoperative differential diagnosis of parotid gland tumors.

## 1. Introduction

The global annual incidence of salivary gland tumors ranges from 0.4 to 13.5 cases per 100,000 people, making up approximately 3–6% of all head and neck tumors [1]. More than 65% of these tumors are benign, with malignant tumors accounting for the remaining 35%. Between 59% and 80% of salivary gland tumors are located within the parotid gland, with the majority located in the superficial lobe. Pleomorphic adenoma is the most frequent benign tumor (60–70%), followed by Warthin tumors (17–30%). With an incidence between 26% and 32.5%, mucoepidermoid carcinoma occupies first place among malignant parotid tumors [1,2,3,4,5].

Surgery is the first therapeutic option for tumors of the parotid gland. Currently, parotidectomy and extracapsular dissection are the main surgical techniques accepted for parotid gland tumors. The main objective of surgical intervention in the case of benign tumors is to ensure local control of the disease while preserving all branches of the facial nerve, thus ensuring minimal morbidity for the patient. In the case of malignant tumors, more extensive surgery respecting oncological principles is required [6,7,8].

The selection of the surgical technique is planned differently depending on the histological type and characteristics of the parotid tumor. Therefore, choosing the surgical procedure that assures the best prognosis requires an accurate preoperative imaging diagnostic that enables the distinction of benign from malignant parotid tumors, as well as adenomas from cystic tumors (Warthin tumors). Accordingly, based on the location, size, and relationship of the tumor with the facial nerve, one may choose parotidectomy or extracapsular dissection for pleomorphic adenomas, while extracapsular dissection is the preferred surgical approach for cystic tumors. In the case of pleomorphic adenomas located in the superficial parotid lobe, with a maximum diameter of up to 3 cm and in contact with the posterior belly of the digastric muscle, extracapsular dissection is the preferred surgical procedure. In cases of pleomorphic adenomas located in the deep parotid lobe or larger than 3 cm, partial or total parotidectomy is indicated because of the risk of damage to the facial nerve. The great advantage of extracapsular dissection is minimal morbidity for the patient, shorter hospitalization time, and reduced risk of Frey’s syndrome or other postoperative complications. In the case of malignant parotid tumors, parotidectomy becomes mandatory [6,7,8].

According to the World Health Organization, over 31 histological subtypes of salivary gland tumors have been identified [1]. The great histological variety and contraindication for biopsy in the parotid region make the accurate preoperative diagnosis of these tumors very challenging for clinicians.

Fine-needle aspiration biopsy (FNAB) provides an alternative to open biopsy and is widely used for parotid tumors. However, due to the frequent overlap of morphological characteristics between histological subtypes of parotid gland tumors, FNAB in this region may lead to a high percentage of undetermined cytological results [9,10].

Multiparametric magnetic resonance imaging (MRI) with diffusion-weighted imaging (DWI) and dynamic contrast-enhanced (DCE) sequences has recently gained recognition as one of the finest and most accurate imaging methods for the preoperative differential diagnosis of salivary gland tumors. Moreover, multiparametric MRI provides the clinician with the most details related to the parotid tumor characteristics, thus significantly influencing the choice of best operative strategy [11,12,13].

Starting with the fact that conventional MRI is the best imaging method for the diagnosis and characterization of parotid tumors, the usefulness of combining conventional and multiparametric MRI in the management of benign and malignant tumors of the parotid gland is unclear because there is currently no consensus in the literature regarding which multiparametric MRI parameters are the most valuable for the preoperative differential diagnosis of these tumors. In order to ensure a predictable differential diagnosis that is simple to perform and less operator-dependent, additional studies are required to determine and validate the usefulness of specific MRI parameters.

There is no consensus in the literature on the best surgical treatment for benign parotid tumors. In recent years, benign parotid tumor surgery has become less invasive, with extracapsular dissection being practiced more often with encouraging outcomes [7,8].

The value and diagnostic accuracy of multiparametric MRI in the preoperative differential diagnosis of parotid gland tumors are still highly debated topics in the literature due to the inadequacies in standardization or significant inter-study variances in outcomes [5,11,14,15,16,17,18,19].

The aim of this study was to determine the value and diagnostic accuracy of multiparametric MRI in the preoperative differential diagnosis of parotid gland tumors (malignant versus benign tumors and pleomorphic adenomas versus Warthin tumors), as well as identify the most significant MRI parameters related to this purpose.

## 2. Materials and Methods

### 2.1. Study Design and Setting

A three-year, single-center, prospective study that began in December 2019 and ended in December 2022 was conducted in our department of maxillofacial surgery. The present study was approved by the ethics committee of the University of Medicine and Pharmacy “Iuliu Hațieganu” Cluj-Napoca, Romania (protocol code 480 from 21 November 2019) and the Emergency Clinical County Hospital, Cluj-Napoca, Romania (protocol code 36752 from 17 December 2019).

### 2.2. Participants

The inclusion criteria included patients with primary tumors of the parotid gland with surgical indications. The exclusion criteria were the following: previously treated tumors or tumor recurrence, patients who refused surgical treatment, lack of surgical indication (inoperable tumors) or general contraindication for surgery, other parotid gland pathology, and patients with contraindications for MRI imaging investigation. After applying the inclusion and exclusion criteria, 65 patients were included in the study. Informed consent was obtained from all patients included in our study.

### 2.3. Data Sources Measurement

Each patient included in the study was investigated preoperatively with the help of a multiparametric MRI, after which an imaging diagnosis was established. Based on this imaging examination, the type of surgical intervention was decided, with each patient undergoing surgical excision of the parotid gland tumor. After the surgical intervention, the preoperative imaging diagnosis was compared with the final histopathological report.

MRI imaging was performed using a Signa Explorer 1.5 T General Electric (GE) system with a dedicated, 16-channel, phased array neuro-vascular coil.

Image analysis was performed by a single radiologist with expertise in MRI imaging and over 15 years of experience in this field. Several MRI parameters were analyzed as follows: signal intensity on T1 and T2 weighted image (WI), apparent diffusion coefficient values (ADC), time-intensity curve (TIC), and time to peak (TTP).

The histopathological analysis of the excised specimen was performed by a single pathologist with over 15 years of expertise in this field.

### 2.4. Statistical Methods

Categorical data were presented as absolute frequencies and percentages. Quantitative data were presented as medians and interquartile ranges and swarm plots combined with boxplots were presented for data not following a normal distribution. Comparisons between two independent groups of categorical data were performed using the Chi-squared test or Fisher’s exact test, where appropriate, while the Wilcoxon rank-sum test was used for skewed quantitative data. Receiver operator characteristic (ROC) plots along with area under the ROC curves with 95% confidence intervals were obtained. The comparison between two ROC curves was performed using the De Long test. The best cut-off point was identified in order to maximize the Youden index and the sensitivity and specificity were computed. The diagnostic accuracy of multiparametric MRI as an index test compared to histopathological examination for malignant versus benign nature of the tumors was assessed using a contingency table, followed by computing sensitivity, specificity, positive predictive value, negative predictive value, positive likelihood ratio, negative likelihood ratio, diagnostic odds ratio, Youden index, and accuracy, with 95% confidence intervals. For all statistical tests, the two-tailed *p*-value was computed and a value of 0.05 was considered as the level of statistical significance. All statistical analyses were carried out in R version 4.1.2 (R Foundation for Statistical Computing, Vienna, Austria) [20].

## 3. Results

### 3.1. Patient Demographics and Tumors Characteristics

A total of 65 patients with primary parotid tumors confirmed by MRI imaging were included in this study. Among these patients, 56.9% (37) were women and 43.1% (28) were men, with a median age of 54 years and range of 47–65 years. The distribution of demographic data of the patients as well as some relevant clinical and imaging characteristics of the benign and malignant parotid tumors are described in Table 1. Univariate statistical analysis showed significant differences among the characteristics of benign and malignant parotid tumors. Patients with malignant parotid tumors were of higher age, lived more frequently in a rural setting, more frequently had deep lobe tumor localization within the parotid gland, had larger tumor sizes, and had longer times from onset to presentation than patients with benign tumors.

### 3.2. Tumors Distribution According to the Histopathologic Subtype

Histopathologic diagnosis was established based on the specimen obtained after surgical resection of the parotid tumor. Among the parotid tumors, 83% (54) were benign and the remaining 17% (11) were malignant. The detailed distribution of the histopathologic subtypes for the 65 parotid gland tumors included in the present study is presented in Table 2.

### 3.3. Multiparametric Magnetic Resonance Imaging Evaluation—Benign versus Malignant Parotid Tumors

A comparative analysis of the MRI parameters used in the differential diagnosis between the benign malignant parotid tumors was performed. This analysis included all 65 parotid tumors. The T2 weighted image signal and apparent diffusion coefficient parameters were statistically significant in differentiating benign from malignant tumors. In the case of the T2WI sequences, mixed signal intensity was found in half of the benign tumors (27) and 63.64% (7) of the malignant tumors presented predominantly hypointense signals. The median ADC value for benign tumors was 1.03 × 10^−^^3^ mm^2^/s and lower than that for malignant tumors, where it was 0.78 × 10^−^^3^ mm^2^/s. No statistically significant results were obtained for the other parameters included in this analysis. Table 3 contains a detailed description of the distribution of the studied parameters.

The diagnostic performance of multiparametric MRI in differentiating benign from malignant parotid tumors, after comparing the preoperative imaging diagnosis with the postoperative histopathological result, was as follows: sensitivity = 81.8% (95% CI 53.2–96.3%), specificity = 88.6% (95% CI 88.6–97.4%), positive predictive value = 75% (95% CI 48.8–88.2%), negative predictive value = 96.2% (95% CI 90.3–99.2%), and accuracy = 92.3% (95% CI 82.6–97.2%).

**Figure 1:**
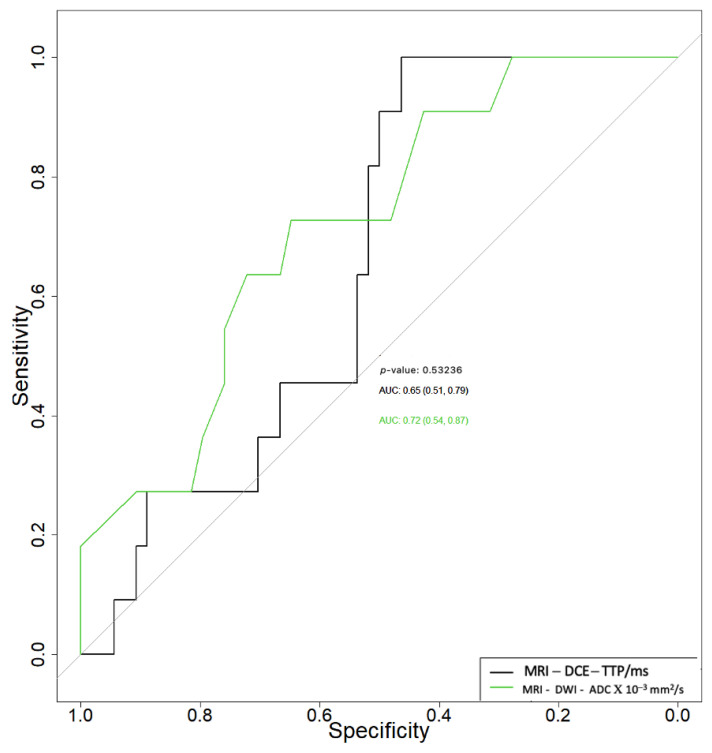
Receiver operating characteristic curves of apparent diffusion coefficient (ADC) and time to peak (TTP) in the differential diagnosis between benign and malignant parotid tumors.

Related to the differential diagnosis between benign and malignant tumors, the ADC and TTP parameters had area under the curve (AUC) values of 0.72 (95% CI 0.54–0.87) and 0.65 (95% CI 0.51–0.79), respectively (Figure 1), with no statistically significant differences between the two (*p* = 0.532). The ADC parameter had a specificity (Sp) and sensitivity (Se) of 65% and 75%, respectively, with a cut-off of 0.88, while the TTP parameter had a specificity and sensitivity of 40% and 100%, respectively, for a cut-off of 112.25 to differentiate between benign and malign tumors.

### 3.4. Multiparametric Magnetic Resonance Imaging Evaluation—Pleomorphic Adenoma versus Warthin Tumor

The diagnostic performance of multiparametric MRI in the differential diagnosis of pleomorphic adenomas from Warthin tumors was analyzed. In this sense, a detailed statistical analysis was conducted including all of the MRI parameters used in this study (Table 4). This analysis was performed on a group of 48 tumors, of which 29 were Warthin tumors and 19 were pleomorphic adenomas. All five parameters—T1WI, T2WI, TIC curves, TTP, and ADC—were statistically significant in discriminating pleomorphic adenomas from Warthin tumors.

Multiparametric MRI associated with morphological analysis by conventional MRI presented a sensitivity, specificity, and accuracy of 94.1% (95% CI 77.8–94.1), 100% (95% CI 90.4–100%), and 97.8% (95% CI 85.7–97.8%), respectively, in the differential diagnosis of pleomorphic adenomas from Warthin tumors. This analysis was performed on 19 pleomorphic adenomas and 29 Warthin tumors, following the exclusion of the other tumor histological types included in the present study—malignant tumors, other types of parotid gland adenomas, and parotid gland cysts.

**Figure 2:**
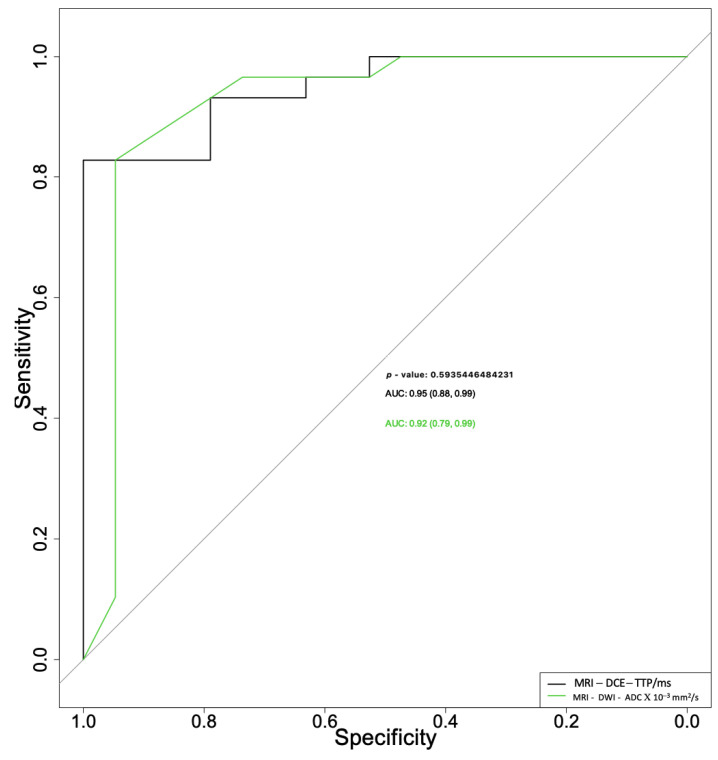
Receiver operating characteristic curves for apparent diffusion coefficient (ADC) and time to peak (TTP) parameters used for differential diagnosis between pleomorphic adenomas and Warthin tumors.

When used to differentiate pleomorphic adenomas from Warthin tumors, the ADC and TTP parameters had area under the curve (AUC) values of 0.92 (95% CI 0.79–0.99) and 0.95 (95% CI 0.88–0.99), respectively (Figure 2), with no statistically significant differences between the two (*p* = 0.594).

The ADC parameter alone presented a sensitivity of 83% and specificity of 95%, with a cut-off of 1.08, and the TTP parameter alone showed a sensitivity of 83% and specificity of 100%, with a cut-off of 1.29.

## 4. Discussion

This prospectively designed study successfully managed to assess the diagnostic accuracy of multiparametric MRI to distinguish between benign and malignant parotid tumors and between pleomorphic adenomas and Warthin tumors, respectively.

Our study results revealed statistically significant differences between benign and malignant parotid tumors in terms of patient age, living environment, tumor location at the level of the parotid gland (deep versus superficial lobe), maximum tumor size, and tumor evolution period. Thus, in line with the findings of other studies, patients with malignant tumors had a greater median age than those with benign tumors (67 versus 54 years) [2,3,21].

The deep parotid lobe was affected by a considerable percentage of malignant tumors (45.4%), which also had a greater median maximum tumor size than benign tumors (37.5 mm versus 25 mm) and a longer evolution period. All of these findings concurred with the data reported by other studies [22].

Regarding the distribution of histological subtypes, the results of our study placed Warthin tumors as occupying the first place among benign parotid tumors (53.7%), followed by pleomorphic adenomas (35.18%). This result disagrees with the majority of studies published in the literature [1,2,3,4]. However, Psychogios et al. reported findings that were comparable with ours, showing a 42.4% incidence of Warthin tumors [23]. Moreover, some studies in the literature claim that there is presently an increasing trend in the incidence of Warthin tumors [17,23,24]. In accordance with the data in the literature, mucoepidermoid carcinomas were the most common malignant tumors in our study (18.18%).

The results of our study demonstrated the diversity of parotid tumor histology; 15 distinct types of tumor histology were found in a group of 65 parotid tumors [1,2,3,4].

A statistical analysis of the multiparametric MRI characteristics used to distinguish between benign and malignant parotid tumors is shown in Table 3. Two parameters—T2WI signal intensity and ADC value—were found to have statistically significant results in this regard. In light of this, when the T2WI MRI sequences were analyzed, hyperintense or mixed signals (predominantly hyperintense signals with areas of hypointense signals) were indicative of benign parotid tumors (81.48% of the benign tumors included in the study), whereas hypointense or mixed signals (predominantly hypointense signals with areas of hyperintense signals) were indicative of malignant parotid tumors (100% of the malignant tumors analyzed in the study), with a *p*-value of 0.004. When using T2WI sequences to differentiate between benign and malignant parotid tumors, Fahem et al. and Christe et al. came to similar conclusions. The study by Faheem et al. showed that 50% of benign tumors presented a hyperintense T2WI signal and 62.5% of malignant tumors presented a hypointense T2WI signal, with a statistically significant *p*-value of 0.032 [22,25].

According to the findings of our study, benign tumors had a median ADC value of 1.03 × 10^−3^ mm^2^/s (IQR: 0.8–1.44), while malignant tumors had a substantially lower median ADC value of 0.78 × 10^−3^ mm^2^/s (IQR—0.68–0.99), indicating a statistically significant difference in the distinction between benign and malignant parotid tumors (*p*-value = 0.024). Karaman et al. obtained similar results in a previous study regarding the mean value of the ADC parameter, with 1.6 × 10^−3^ mm^2^/s for benign tumors and 0.8 ± 0.3 × 10^−3^ mm^2^/s for malignant tumors [26]. On the other hand, other authors did not find a statistically significant difference between the ADC values of benign and malignant parotid tumors due to the overlap of ADC values between Warthin tumors and malignant tumors such as lymphoma. This was attributed to the densely compacted lymphoid tissue characteristic of Warthin tumors, which can cause confusion with malignant tumors [15]. As a result, the use of several MRI parameters in addition to the ADC value, such as TIC curves and TTP, can contribute to increasing the accuracy of preoperative diagnosis.

The power of two MRI parameters (T2WI and ADC) to discriminate between benign and malignant parotid tumors has been observed in other studies published in the literature [17,18,22,26,27,28].

In our study, a significant number of benign tumors showed type A or B TIC curves (62.96%), whereas 45.45% of malignant tumors showed a type C TIC curve, despite the fact that the TIC parameter did not prove to be statistically significant (*p* = 0.382) in the differentiation of benign from malignant parotid tumors. There is currently agreement in the literature that type A and B TIC curves are specific to benign tumors, while a type C TIC curve is indicative of a malignant parotid tumor [12,29,30,31].

**Figure 3:**
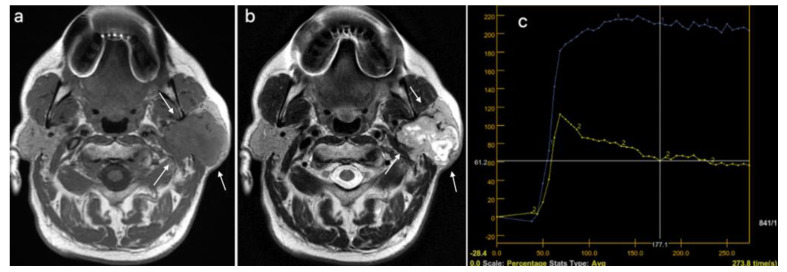
Pleomorphic adenoma of the left parotid gland (white arrows): (**a**) T1WI sequence showing a hypointense signal in the lobulated lesion, well delineated; (**b**) T2WI sequence showing an inhomogeneous, predominantly hyperintense signal in the tumor, with central cystic areas; (**c**) DCE-MRI showing a type A TIC curve.

**Figure 4:**
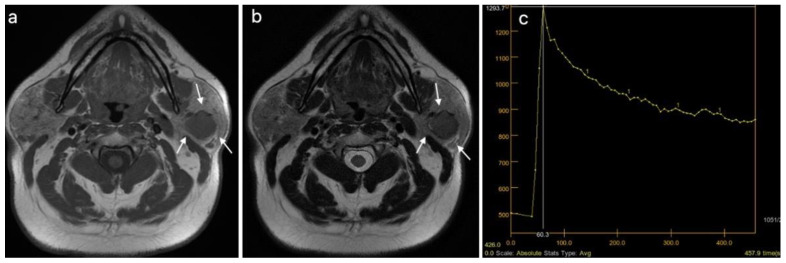
Warthin tumor of the left parotid gland (white arrows): (**a**) T1WI sequence showing a hypointense signal in the tumor, well delineated, with slightly irregular borders; (**b**) T2WI sequence showing a predominantly hypointense signal in the lesion, with peripheral cystic area; (**c**) DCE-MRI showing a type B TIC curve.

**Figure 5:**
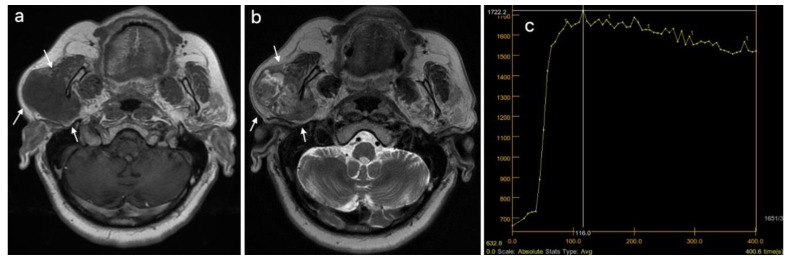
Malignant tumor of the right parotid gland (white arrows): (**a**) T1WI sequence showing an inhomogeneous hypointense signal in the tumor, with infiltrative borders; (**b**) T2WI sequence also showing an inhomogeneous signal, with central necrotic areas; (**c**) DCE-MRI showing a type C TIC curve.

Three different patients, diagnosed using multiparametric MRI, with a pleomorphic adenoma, a Warthin tumor, and a malignant tumor of the parotid gland are presented in Figure 3, Figure 4 and Figure 5.

All conventional MRI characteristics (T1WI and T2WI) and multiparametric MRI measurements (TIC curve, TTP, and ADC value) utilized in this study were found to be statistically significant in the distinction between pleomorphic adenomas and Warthin tumors. A *p*-value <0.001 was obtained for the T2WI, TIC, TTP, and ADC parameters, and a *p*-value **=** 0.024 was obtained in the case of the T1WI parameter. Therefore, we can assume that a hypointense signal (78.9% of cases) when evaluating T1WI sequences is specific to a pleomorphic adenoma.

Contrary to Warthin tumors, which exhibit mixed hypointense T2WI signals (hypointense signals with hyperintense signal areas) or hypointense signals in 89.66% of cases, pleomorphic adenomas exhibit characteristic hyperintense or mixed signals (predominantly hyperintense signals with hypointense signal areas) in a high proportion of 94.74% of cases. Numerous experts in the field agree that the “bright signal” (a hyperintense signal in T2WI) is a crucial criterion for the differential diagnosis of pleomorphic adenomas using conventional MRI [12,26,27,32,33].

The findings from our study demonstrated that using DCE-MRI analysis, 78.94% of pleomorphic adenomas displayed a type A TIC curve, which agreed with similar studies [31,32]. Xu et al. reported in their study that pleomorphic adenomas presented an A-type curve in 74% of cases [31].

In 37.93% of cases, Warthin tumors displayed a type B TIC curve, whereas 62.07% of cases displayed a type C TIC curve (*p* < 0.001). In contrast with our results, the type B curve was most frequently encountered in Warthin tumors in other studies [29,31,32]. Regarding the appearance of TIC curves, the possible overlap of the type C TIC curve between Warthin tumors, parotid lymphomas, as well as other malignant parotid tumors is reported in the literature. Most authors agree that for accurate differentiation between these histological types, it is necessary to add other parameters in the MRI analysis [17,29,34,35]. In this regard, T1 and T2 WI, TTP, and ADC value were additional factors in our study that contributed to the differential diagnosis of parotid tumors.

Statistically significant results (*p* < 0.001) were obtained following DCE-MRI analysis of the time to peak (TTP) parameter in differentiating pleomorphic adenomas (median value of 210 ms) from Warthin tumors (median value of 88.5 ms) with high sensitivity (83%) and specificity (100%). Similar results were obtained in other studies that used the TTP parameter in the differentiation of parotid tumors with the help of DCE-MRI [15,26]. Elmokaden et al. reported in their research a much higher TTP value for pleomorphic adenomas (mean value of 185.73 ± 90.66 ms) than for Warthin tumors (mean value of 65.45 ± 80.34 ms) [15].

Regarding the ADC parameter, the results of our study showed that pleomorphic adenomas recorded the highest value (mean value of 1.5 × 10^−3^ mm^2^/s) among parotid tumors. This parameter was statistically significant in differentiating malignant from benign parotid tumors (*p* < 0.024), as well as pleomorphic adenomas from Warthin tumors (*p* < 0.001). The ROC analysis revealed that the ADC parameter had a high AUC value (0.92) and high sensitivity (83%) and specificity (95%) for its best cut-off in distinguishing pleomorphic adenomas from Warthin tumors. Regarding research on the differential diagnostic power of the ADC parameter in parotid gland tumors, many authors came to similar conclusions [17,18,26,27,32,34]. However, in some studies, the ADC parameter was able to distinguish between pleomorphic adenomas and Warthin tumors while failing to distinguish between benign and malignant parotid tumors [15,22]. In our opinion, the correct selection of the most representative region of interest (ROI) of the parotid tumor in multiparametric MRI analysis was particularly crucial for the precise application of this parameter. The ADC values of parotid gland tumors reported by various authors in their studies do, however, differ slightly. Yabuuchi et al. reported mean values for Warthin tumors, pleomorphic adenomas, and malignant tumors of 0.86, 1.92, and 0.8–1.2, respectively [36], while Xu et al. reported mean values for the same tumor types of 1.36, 0.66, and 0.85, respectively [31].

**Figure 6:**
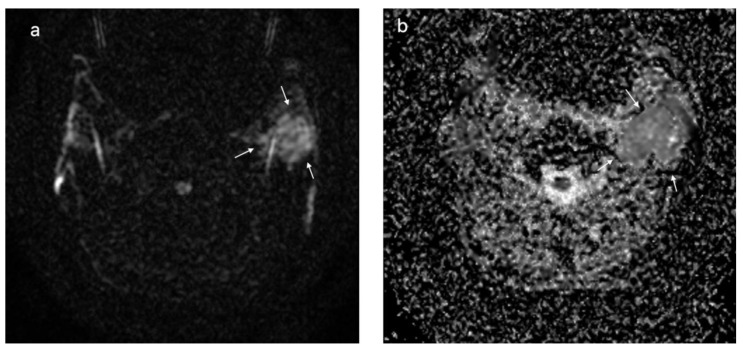
Pleomorphic adenoma located at the level of the left parotid gland (white arrows); DWI sequences (**a**) with ADC map (**b**) showing a value of 1.6 × 10^−^^3^ mm^2^/s.

**Figure 7:**
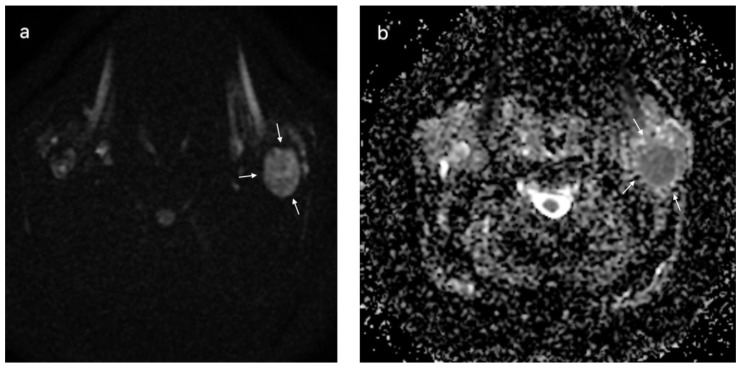
Warthin tumor of the left parotid gland (white arrows); DWI sequences (**a**) with ADC map (**b**) showing a value of 0.72 × 10^−^^3^ mm^2^/s.

**Figure 8:**
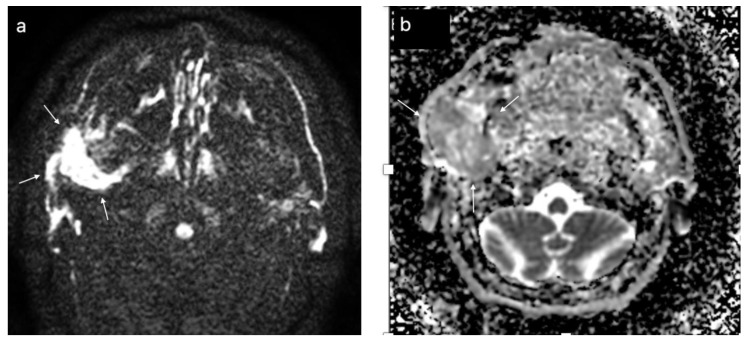
Malignant tumor of the left parotid gland (white arrows); DWI sequences (**a**) with ADC map (**b**) showing a value of 1.1 × 10^−^^3^ mm^2^/s.

In Figure 6, Figure 7 and Figure 8, the DWI sequences with ADC maps are presented for a pleomorphic adenoma, Warthin tumor, and a malignant parotid tumor.

The results obtained in this study show applicability in the preoperative differential diagnosis of parotid tumors in clinical practice. The strength of this paper is represented by its ability to provide clinicians with a workflow for preoperative imaging diagnosis of parotid gland tumors using morphological MRI analysis (T1WI and T2WI) combined with only three DCE and DWI-MRI parameters (TIC, ADC, and TTP), which are straightforward to obtain in an acceptable amount of time for image acquisition and processing. Additionally, to our knowledge, no studies in the literature have combined these five MRI parameters for the differential diagnosis of parotid gland tumors.

The clinical significance of preoperative distinction between benign and malignant parotid tumors is represented by the fact that the tumor histology dictates the surgical treatment. Therefore, a malignant tumor will be treated using a more extensive surgical approach according to oncological principles, whereas a benign tumor can be treated with minimal invasiveness either by extracapsular dissection or parotidectomy, depending on the location, size, and relationship of the tumor to the facial nerve. Furthermore, the preoperative differentiation between Warthin tumors and pleomorphic adenomas allows the surgeon to make a clear selection of cases that lend themselves to a minimally invasive approach, such as extracapsular dissection.

The clinical importance of differentiating Warthin tumors from pleomorphic adenomas resides in the fact that Warthin tumors always require a minimally invasive approach due to the much lower risk of recurrence compared to pleomorphic adenomas. In this sense, an accurate preoperative diagnosis contributes decisively to the choice of extracapsular dissection as the preferred operative technique for these tumors.

Our study had several limitations, including the single-center design, relatively small group of patients (relatively rare pathology), and lack of uniform distribution between the number of benign and malignant parotid tumors (in accordance with the known incidence ratio between them). It should be mentioned that ADC values and TIC curve patterns might differ greatly depending on the hardware, applied methodology, inter-observer variations, or biological variables. For further validation of our findings, a large multicenter study with MRI image analysis conducted by multiple radiologists should be performed and an inter-observer reliability assessment is warranted.

## 5. Conclusions

MRI investigation is essential in the preoperative differential diagnosis of parotid gland tumors and has an important value in the decision of treatment strategy. Morphological analysis using conventional MRI combined with analysis of the DWI and DCE sequences of multiparametric MRI enables the preoperative differential diagnosis of parotid gland tumors. The strongest diagnostic power for differentiating between benign and malignant parotid tumors was provided by the analysis of T2WI sequences from conventional MRI and ADC values obtained with DWI-MRI.

Pleomorphic adenomas and Warthin tumors can be distinguished using conventional T1WI and T2WI MRI sequences, as well as each individual DCE and DWI-MRI parameter, including the TIC pattern, TTP, and ADC value. Combined analysis using conventional and multiparametric MRI ensured a high diagnostic power for differential diagnosis between pleomorphic adenomas and Warthin tumors, with increased rates of accuracy, sensitivity, and specificity.

## Figures and Tables

**Table 1:** Patient demographics and benign and malignant tumor characteristics.

Histopathology:	Benign	Malignant	*p*–Value
	(*n* = 54)	(*n* = 11)	
Age (years), median (IQR)	54 (46–63)	68 (64–69)	0.003
Gender (female), n (%)	32 (58.18)	5 (45.45)	0.509
Living environment (urban vs. rural), n (%)	50 (92.59)	5 (45.45)	<0.001
Tumor localization (left vs. right), n (%)	27 (50)	2 (18.18)	0.094
Tumor localization within the parotid gland (deep lobe vs. superficial lobe), n (%)	9 (16.36)	5 (45.45)	0.031
Maximum tumor size (mm), median (IQR)	25 (19–32.75)	42 (27.5–48.5)	0.029
Time from onset to presentation (months), median (IQR)	12 (6–24)	24 (20–48)	0.015

IQR, interquartile range.

**Table 2:** Histopathologic subtypes of parotid gland tumors.

	Benign	Malignant
	(*n* = 54)	(*n* = 11)
Histopathologic subtype	Warthin tumor: 29 (53.7)	
	Pleomorphic adenoma 19 (35.18)	Mucoepidermoid carcinoma: 2 (18.18)
	Basal cell adenoma: 3 (5.55)	Ductal adenocarcinoma: 1 (9.09)
	Oncocytic papillary cystadenoma: 1 (1.85)	Basal cell carcinoma: 1 (9.09)
	Parotid gland cyst: 2 (3.7)	Acinic cell carcinoma: 1 (9.09)
		Salivary duct carcinoma: 1 (9.09)
		Carcinoma ex pleomorphic adenoma: 1 (9.09)
		Intraductal carcinoma: 1 (9.09)
		Squamous cell carcinoma: 1 (9.09)
		Intraparotid gland melanoma Lymph node metastasis: 1 (9.09)
		Undifferentiated sarcoma: 1 (9.09)

**Table 3:** Multiparametric MRI parameters—comparison between benign and malignant parotid tumors.

MRI Parameters	Histology		
	Benign	Malignant	*p*–Value
	(*n* = 54)	(*n* = 11)	
MRI diagnosis, n (%)			
benign	51 (94.44)	2 (18.2)	
Malignant	3 (5.56)	9 (81.8)	
MRI, T1 weighted image, n (%)			0.272
Hyperintense signal	9 (16.67)	1 (9.09)	
Mixed signal intensity	16 (29.63)	1 (9.09)	
Hypointense signal	29 (53.7)	9 (81.82)	
MRI, T2 weighted image, n (%)			0.004
Hyperintense signal	17 (31.48)	0 (0)	
Mixed signal intensity	27 (50)	4 (36.36)	
Hypointense Signal	10 (18.52)	7 (63.64)	
DCE MRI^*^–TIC curve^+^, n (%)			0.382
Type A	16 (29.63)	1 (9.09)	
Type B	18 (33.33)	5 (45.45)	
Type C	20 (37.04)	5 (45.45)	
DCE MRI, TIC curve grouped (A, B vs. C), n (%)	34 (62.96)	6 (54.5S5)	0.737
DCE MR, TTP (ms), median (IQR)	133.5 (87.15–203.75)	143 (138–204.45)	0.113
DWI MRI, ADC × 10^−3^ mm^2^/s, median (IQR)	1.03 (0.8–1.44)	0.78 (0.68–0.99)	0.024

MRI, magnetic resonance imaging; DCE, dynamic contrast-enhanced; TTP, time to peak; TIC, time-intensity curves; DWI, diffusion-weighted imaging; ADC, apparent diffusion coefficient; IQR, interquartile range.

**Table 4:** Multiparametric MRI parameters—comparison between pleomorphic adenomas and Warthin tumors.

MRI Parameters	Histology		
	Pleomorphic Adenoma	Warthin Tumor	*p*-Value
	*n* = 19	*n* = 29	
MRI, T1 weighted image, n (%)			0.024
Hyperintense signal	1 (5.26)	7 (24.14)	
Mixt signal intensity	3 (15.79)	11 (37.93)	
Hypointense signal	15 (78.95)	11 (37.93)	
MRI, T2 weighted image, n (%)			<0.001
Hyperintense signal	12 (63.16)	3 (10.34)	
Mixed signal intensity	6 (31.58)	20 (68.97)	
Hypointense signal	1 (5.26)	6 (20.69)	
DCE MRI, TIC curve, n (%)			<0.001
Type A	14 (73.68)	0 (0)	
Type A, C	1 (5.26)	0 (0)	
Type B	3 (15.79)	11 (37.93)	
Type C	1 (5.26)	18 (62.07)	
DCE MRI, TTP (ms), median (IQR)	210 (166.15–228.9)	88.5 (82–101.9)	<0.001
DWI MRI, ADC × 10^−3^ mm^2^/s, median (IQR)	1.5 (1.25–2.1)	0.86 (0.73–1)	<0.001

MRI, magnetic resonance imaging; DCE, dynamic contrast-enhanced; TTP, time to peak; TIC, time-intensity curves; DWI, diffusion-weighted imaging; ADC, apparent diffusion coefficient; IQR, interquartile range.

## Data Availability

The data presented in this study are available in this article.
